# TGFBR3L is associated with gonadotropin production in non-functioning gonadotroph pituitary neuroendocrine tumours

**DOI:** 10.1007/s11102-023-01310-x

**Published:** 2023-03-23

**Authors:** Anders Jensen Kolnes, Kristin Astrid Berland Øystese, Evelina Sjöstedt, Nicoleta Cristina Olarescu, Ansgar Heck, Jens Pahnke, Daniel Dahlberg, Jon Berg-Johnsen, Geir Ringstad, Olivera Casar-Borota, Jens Bollerslev, Anders Palmstrøm Jørgensen

**Affiliations:** 1 Department of Medical Biochemistry, Institute of Clinical Medicine, Faculty of Medicine, University of Oslo, Sognsvannsveien 20, 0372 Oslo, Norway; 2 Section of Specialized Endocrinology, Department of Endocrinology, Oslo University Hospital, Oslo, Norway; 3 Department of Neuroscience, Karolinska Institutet, Stockholm, Sweden; 4 Department of Immunology, Genetics and Pathology, Uppsala University, Uppsala, Sweden; 5 Department of Pathology, Section of Neuropathology, University of Oslo and Oslo University Hospital, Oslo, Norway; 6 Pahnke lab (Drug Discovery and Chemical Biology), Lübeck Institute of Dermatology, LIED, University of Lübeck, Lübeck, Germany; 7 Department of Pharmacology, Faculty of Medicine, University of Latvia, Rīga, Latvia; 8 Department of Neurosurgery, Oslo University Hospital, Oslo, Norway; 9 Department of Radiology, Oslo University Hospital, Oslo, Norway; 10 Department of Clinical Pathology, Uppsala University Hospital, Uppsala, Sweden

**Keywords:** Pituitary adenomas, Plasma gonadotropins, Tumour volume, Transforming growth factor beta receptor 3 like (TGFBR3L), Transforming growth factor beta receptor 3 (TGFBR3)

## Abstract

**Purpose:**

Transforming growth factor-beta receptor 3-like (TGFBR3L) is a pituitary enriched membrane protein selectively detected in gonadotroph cells. TGFBR3L is named after transforming growth factor-beta receptor 3 (TGFBR3), an inhibin A co-receptor in mice, due to sequence identity to the C-terminal region. We aimed to characterize TGFBR3L detection in a well-characterized, prospectively collected cohort of non-functioning pituitary neuroendocrine tumours (NF-PitNETs) and correlate it to clinical data.

**Methods:**

144 patients operated for clinically NF-PitNETs were included. Clinical, radiological and biochemical data were recorded. Immunohistochemical (IHC) staining for FSHβ and LHβ was scored using the immunoreactive score (IRS), TGFBR3L and TGFBR3 were scored by the percentage of positive stained cells.

**Results:**

TGFBR3L staining was selectively present in 52% of gonadotroph tumours. TGFBR3L was associated to IRS of LHβ (median 2 [IQR 0–3] in TGFBR3L negative and median 6 [IQR 3–9] in TGFBR3L positive tumours, p < 0.001), but not to the IRS of FSHβ (p = 0.32). The presence of TGFBR3L was negatively associated with plasma gonadotropin concentrations in males (P-FSH median 5.5 IU/L [IQR 2.9–9.6] and median 3.0 [IQR 1.8–5.6] in TGFBR3L negative and positive tumours respectively, p = 0.008) and P-LH (median 2.8 IU/L [IQR 1.9–3.7] and median 1.8 [IQR 1.1-3.0] in TGFBR3L negative and positive tumours respectively, p = 0.03). TGFBR3 stained positive in 22% (n = 25) of gonadotroph tumours with no correlation to TGFBR3L.

**Conclusion:**

TGFBR3L was selectively detected in half (52%) of gonadotroph NF-PitNETs. The association to LHβ staining and plasma gonadotropins suggests that TGFBR3L may be involved in hormone production in gonadotroph NF-PitNETs.

**Supplementary Information:**

The online version contains supplementary material available at 10.1007/s11102-023-01310-x.

## Significance Statement

Transforming growth factor-beta receptor 3-like (TGFBR3L), is a pituitary specific membrane protein in gonadotroph cells, named after transforming growth factor-beta receptor 3 (TGFBR3) due to a sequence identity to the C-terminal region.

We describe that TGFBR3L is present at protein level in half of gonadotroph NF-PitNETs.

The relation between TGFBR3L and gonadotropin expression and plasma levels may suggest a role in gonadotropin production and secretion.

The lack of an association to cavernous sinus invasiveness, and lack of consecutive MRI follow-up data limits the conclusion concerning the potential role in tumour growth and aggressiveness.

In human gonadotroph NF-PitNETs, the role of TGFBR3, a known inhibin A co-receptor in mice, appears to be limited since its detection is not associated to gonadotropins measurements, nor to the TGFBR3L expression.

## Introduction

Pituitary neuroendocrine tumours (PitNETs), also known as pituitary adenomas, can arise from any of the cell lineages of the anterior pituitary gland, and can be clinically functioning or non-functioning [1–3]. Per definition, clinically non-functioning PitNETs (NF-PitNETs) do not cause signs or symptoms of hormone hypersecretion [4]. Still, the tumours may cause symptoms due to local mass effect, such as hypopituitarism, visual disturbances, and headache. Surgery is the treatment of choice, but usually delayed until vision is affected [4, 5].

Clinically NF-PitNETs arise most commonly from the gonadotroph cells (~ 73%) [1, 3, 6]. NF-PitNETs are classified by immunohistochemical (IHC) staining for pituitary hormones and cell-lineage specific pituitary transcription factors. Transcription factors were recently introduced into the classification of PitNETs and allow the characterization of hormone-negative tumours [1, 3].

The majority of gonadotroph PitNETs produce gonadotropins, but they rarely cause clinical symptoms of hormone hypersecretion and are thus considered non-functioning [7, 8]. Still, patients with gonadotroph PitNETs frequently have elevated plasma levels of the gonadotropins (FSH and LH) or their subunits [9–13]. Furthermore, most gonadotroph PitNETs secrete gonadotropins or gonadotropin subunits when cultured in vitro [11, 14, 15]. We have recently shown that FSHβ staining of the gonadotroph PitNETs correlated with circulating plasma FSH levels, suggesting that some of the FSH produced by the tumours enters the circulation. However, we found no correlation between LHβ staining and circulating plasma LH levels [16]. Indeed, both in vivo and in vitro, FSH or FSHβ appears to be secreted more commonly than LH or LHβ [9, 11–13]. The regulation of gonadotropin synthesis and secretion is complex and involves GnRH, inhibins, activins, follistatin and moreover negative feedback from estradiol and testosterone [17, 18]. Activins and inhibins belong to the TGF-β superfamily, and are involved in numerous processes throughout the body. The production and secretion of FSHβ and LHβ is stimulated by the pulsatile secretion of GnRH from the hypothalamus, through the GnRHR receptor, in concert with effects of activins in pituitary gonadotroph cells [17, 18]. The production is negatively regulated through negative endocrine feedback mechanisms from the ovaries in females and testicles in males (estrogen, inhibin B, progesterone and testosterone) [19]. In addition, the expression and secretion of gonadotropins are regulated by paracrine and autocrine mechanisms through follistatin, activins and their respective receptors and coreceptors. The interplay between the different participants of the gonadotroph regulation is complex and not fully characterized in humans [17].

Transforming growth factor beta receptor 3 like (TGFBR3L) is a transmembrane protein selectively expressed in pituitary gonadotroph cells [20–24]. TGFBR3L shows sequence similarity to transforming growth factor beta-receptor 3 (TGFBR3), also called betaglycan, that has been detected in rodent pituitary gland and is suggested as an inhibin A co-receptor [24, 25]. Recently, TGFBR3L was shown to function as an inhibin B co-receptor in mice, as TGFBR3L knockout female mice presented a modest increase in FSH levels that was sufficient to enhance ovarian folliculogenesis and litter size [23].

Recently, we found that TGFBR3L correlated positively with FSHβ and LHβ protein content in neoplastic gonadotroph cells [26]. In addition, TGFBR3L staining was associated with lower levels of membranous E-cadherin, a well-known marker of epithelial to mesenchymal transition (EMT). Loss of membranous E-cadherin, predicts a more aggressive behaviour in many tumour types, though this has not consistently been shown for gonadotroph NF-PitNETs [27–30].

In this prospective study, we aimed to:

1. Validate the results from our previous retrospective study showing that TGFBR3L was expressed selectively in gonadotroph NF-PitNETs, and that the staining correlated positively with FSHβ/LHβ staining.
2. Investigate the relationship between TGFBR3L staining and clinical data from patients with clinically NF-PitNETs, including tumour size and invasiveness, and levels of circulating plasma gonadotropins.
3. Investigate whether TGFBR3L staining was associated with TGFBR3 staining in the gonadotroph PitNETs.

## Materials and methods

One hundred and forty four patients of a total of 163 patients operated for NF-PitNETs were included in this prospective study. Before surgery, the tumours were characterized clinically and biochemically as non-functioning. The inclusion criteria were (i) patients undergoing surgery for clinically NF-PitNET; (ii) no previous surgery or radiation of the pituitary gland; and for this particular investigation (iii) available paraffin embedded tissue. Nineteen patients were excluded due to (i) slides unavailable for IHC (n = 7); (ii) necrotic tissue samples (n = 6); (iii) immunohistochemical (IHC) diagnosis other than PitNET (n = 3); and (iiii) no tumour tissue visible on the slides (n = 3). All operations were performed by four neurosurgeons between December 2014 and September 2020, at the Department of Neurosurgery, Oslo University Hospital. The transsphenoidal approach was used in all except three patients. The diagnosis of PitNET was confirmed by a specialist in neuropathology. The study was approved by the Regional Ethics Committee (REK number 2014/1680 approved 23.10.2014). All patients agreed to participate in the study and provided written informed consent.

Classification of PitNET subtype was determined by IHC staining for anterior pituitary hormones and for the pituitary transcription factors in a subset of patients (n = 52). In tumours operated before June 2019, staining for pituitary transcription factors (SF-1, T-Pit, Pit-1) was only performed if the subtype could not be determined based on pituitary hormones alone. Thereafter, transcription factors were routinely investigated in all patients.

We used formalin fixed paraffin embedded tissue and routine IHC staining procedures with Ventana machines (Roche). The following primary antibodies were used: T-Pit (Abcam ab243028, 1:1,000, clone: CL6251), SF-1 (Abcam ab217317, 1:2,000, clone: EPR19744), Pit-1 (Santa Cruz Biotechnology sc-393,943, 1:200, clone: D-7), LHβ (ThermoFisher Scientific MA5-12138, 1:2,000, clone: LH01), FSHβ (ThermoFisher Scientific PA5-111750, 1:200, polyclonal), and ACTH (Agilent (DAKO) M350101-2, 1:200, clone: 02A3). TGFBR3L (Atlas antibodies HPA074356, 1:200, polyclonal) and TGFBR3 (Origene, 2703.00.02, 1:2000, polyclonal) were both stained and validated within the Human Protein Atlas pipeline at Uppsala University using Autostainer XL (Leica, ST5010) for section preparation and Autostainer 480 (ThermoFisher Scientific) including UltraVision™ Quanto Detection System protocol for IHC staining.

IHC staining for FSHβ and LHβ (performed by OC-B) was scored using the immunoreactivity score (IRS) [31]. IRS is the product of the percentage of positive staining cells (0 = 0% positive cells; 1 = 1–10%; 2 = 10–50%; 3 = 50–80%; 4 ≥ 80%) multiplied by the intensity of staining (0 = no staining; 1 = weak staining; 2 = moderate staining; 3 = strong staining), and ranges from 0 to 12 (exceptions 5, 7, 10 and 11) (Supplementary figure). TGFBR3L (performed by ES) and TGFBR3 (OC-B) staining was scored from 0 to 3 (negative: no positive cells; low: 1–10% positive cells; moderate: 10–30% positive cells; high: ≥30% positive cells). Whole tissue sections were used for the IHC evaluation. The reason for using the 4-category scoring system for TGFBR3L and TGFBR3 was that the vast majority of positive tumours showed strong expression of these markers in ≤ 10% positive cells. Using the IRS scoring system would result in IRS score 3 in almost all positive tumours. O.C.B. and E.S. were responsible for the IHC scoring and both were blinded to clinical data. TGFBR3L scoring was missing from one patient, and TGFBR3 scoring was missing from three patients.

Preoperatively, patients underwent clinical and radiological work-up. None of the patients had signs or symptoms of hormone overproduction. Blood samples were collected prior to surgery and analysed by routine laboratory methods. Blood samples were available from 141 patients, and information on preoperative plasma FSH and LH was available in 113 and 114 patients with gonadotroph tumours, respectively. Patients’ medical records were reviewed in retrospect to determine the use of estrogen/testosterone and information on menstrual cycle prior to the primary pituitary surgery. Sixteen of the men received testosterone substitution therapy prior to surgery. Three men received anti-androgen treatment due to prostate disease. In menstruating women, the timing of blood samples according to the menstrual cycle was not available. In cases where the time of menopause or menstrual cycle were not described in the patient record, women aged above 51 years were considered postmenopausal, in accordance with the average age for menopause in Norway [32].

MR images were investigated to determine tumour size and cavernous sinus invasiveness. Size was determined by measuring height, width and depth of the pituitary lesion. The volume was calculated using the formula for an ellipsoid 4/3 x π x (height/2) x (width/2) x (depth/2). Knosp score was used to evaluate invasiveness of the cavernous sinus [33, 34]. Tumours with a Knosp score ≥ 3 were considered invasive. Examination of MR images was performed by A.J.K. under the supervision of G.R.

Statistical analyses for group comparisons were performed with Chi square for nominal data and with Mann-Whitney *U-*test for continuous data. Spearman rank correlation coefficient was used for correlation analyses. A p-value < 0.05 was considered significant. Statistical analyses were performed using Stata version 16.1 (StataCorp LLC, College Station, Texas) and SPSS Version 28.

## Results

### Clinical and IHC characteristics of the patients

One hundred forty four patients were included in the study (females 42%, n = 61). The median age at the time of surgery was 61 years (interquartile range [IQR] 50–70 years) for the whole cohort and 62 years (IQR 54–72) year for the gonadotroph group. IHC staining characterized 80.6% (n = 116) of the cohort as gonadotroph NF-PitNETs (Table 1), of these 44 (38%) female. Of the gonadotroph tumours twelve did not stain (IRS 0) for neither FSHβ nor LHβ, six stained only FSHβ, 28 stained for LHβ alone and 70 for both FSHβ and LHβ. All the tumours were macroadenomas and 30% presented a Knosp score ≥ 3 (27% in the gonadotroph group). The main indication for surgery was visual disturbances.

**Table 1:** Baseline characteristics

Sex	Female 42% (n = 61) Male 58% (n = 83)
**Age**	61 years (IQR: 50–70) • Females 61 (IQR 47–70) • Males 61 (IQR 52–70)
**Tumour volume (mm** ^**3**^ **)**	6224 (IQR: 3876–9646)
**Invasive (Knosp ≥ 3)** **Non-invasive (Knosp ≤ 2)**	30.6% (n = 44) 69.4% (n = 100)
**Indication for surgery** • Visual disturbances • Tumour growth • Headache • Apoplexy	87.5% (n = 126) 10.3% (n = 15) 0.7% (n = 1) 1.4% (n = 2)
**PitNET subtype** • Gonadotroph • Corticotroph • Plurihormonal Pit-1 tumours • Somato-lactotroph • Plurihormonal (SF-1 and Pit-1) • Double PitNET • Hormone and transcription factor negative	80.6% (n = 116) 13.2% (n = 19) 1.4% (n = 2) 1.4% (n = 2) 0.7% (n = 1) 0.7% (n = 1) 2.0% (n = 3)

Numbers are given as percentages or median with interquartile range (IQR).

### Immunohistochemical staining of transforming growth factor beta receptor 3 like protein

**Fig. 1:**
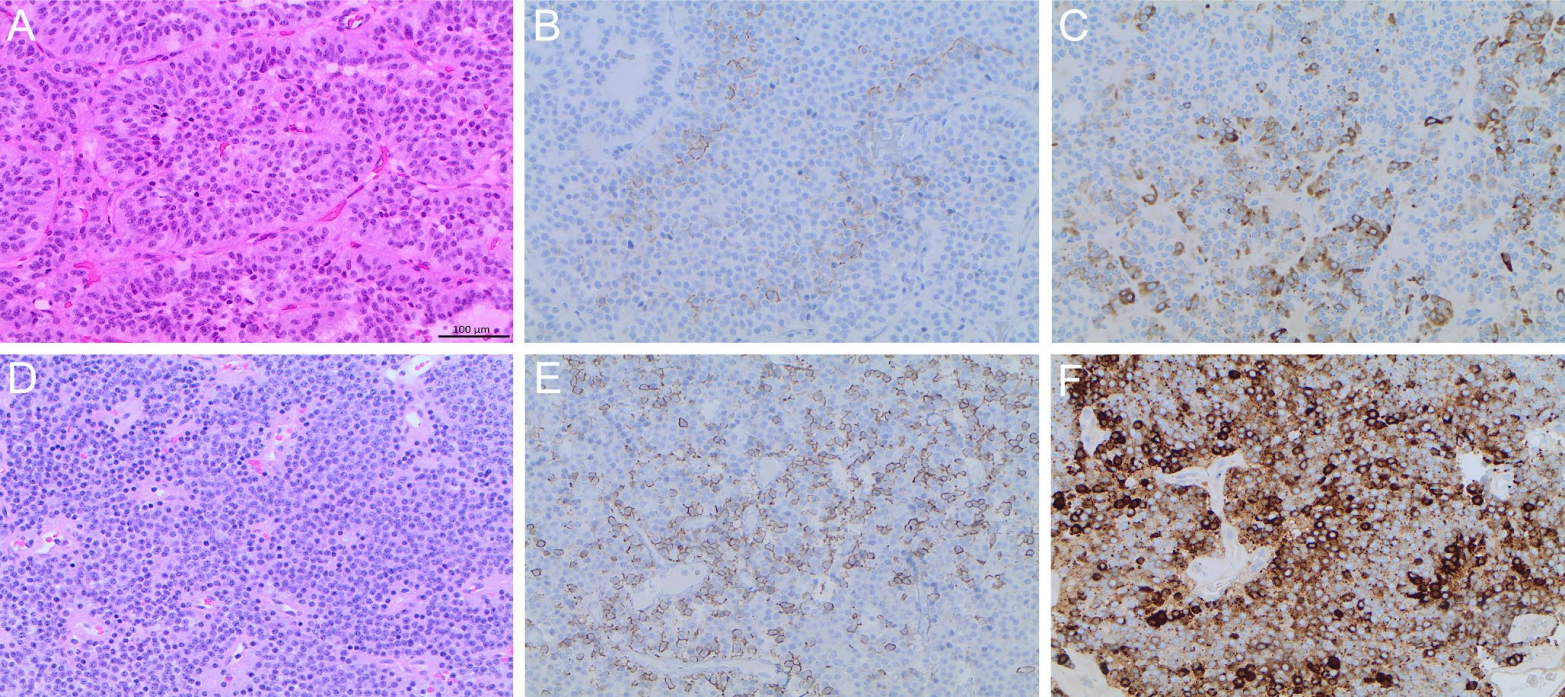
**Immunohistochemical staining of TGFBR3L and LHβ in two pituitary tumours A-C and D-F**. Hematoxylin and eosin, and immunohistochemical stainings of two gonadotroph pituitary tumours (PitNETS). Hematoxylin and eosin staining (A and D); TGFBR3L staining low (1–10% positive cells) and high (≥ 30% positive cells) (B and E); and their corresponding LHβ IRS score 6 and 9 (C and F)

TGFBR3L staining was present in 52% (n = 60) of the gonadotroph tumours, and in one double-PitNET (positive for FSHβ and SF-1 in some cells, and for ACTH and T-Pit in others), while all other tumours were negative. The results of TGFBR3L staining in gonadotroph tumours are shown in Table 2. TGFBR3L staining was similar in males (n = 34, 48% positive) and females (n = 26, 59% positive, p = 0.2), and was not associated with the age of the patients. Only four tumour samples presented between 10 and 30% (n = 3) or ≥ 30% cells (n = 1) positive for TGFBR3L, and for the purpose of further statistical analyses, we decided to dichotomize into negative and positive tumours. Figure 1 shows hematoxylin and eosin (A,D) and IHC staining for TGFBR3L in a patient with low (1–10% positive cells) (B) and in a patient with high (E) (≥ 30% positive cells) proportion of positive cells and their corresponding IRS LHβ score of 6 (C) and 9 (F).

**Table 2:** Percentage TGFBR3L-positve cells in gonadotroph PitNETs*

TGBR3L staining	Percentage of tumours
Negative	47.8% (N = 55)
≤ 10% positive cells	48.7% (N = 56)
10–30% positive cells	2.6% (N = 3)
≥ 30% positive cells	0.9% (N = 1)
Total	N = 115

*One tumour was not available with IHC staining for TGFBR3L.

**Fig. 2:**
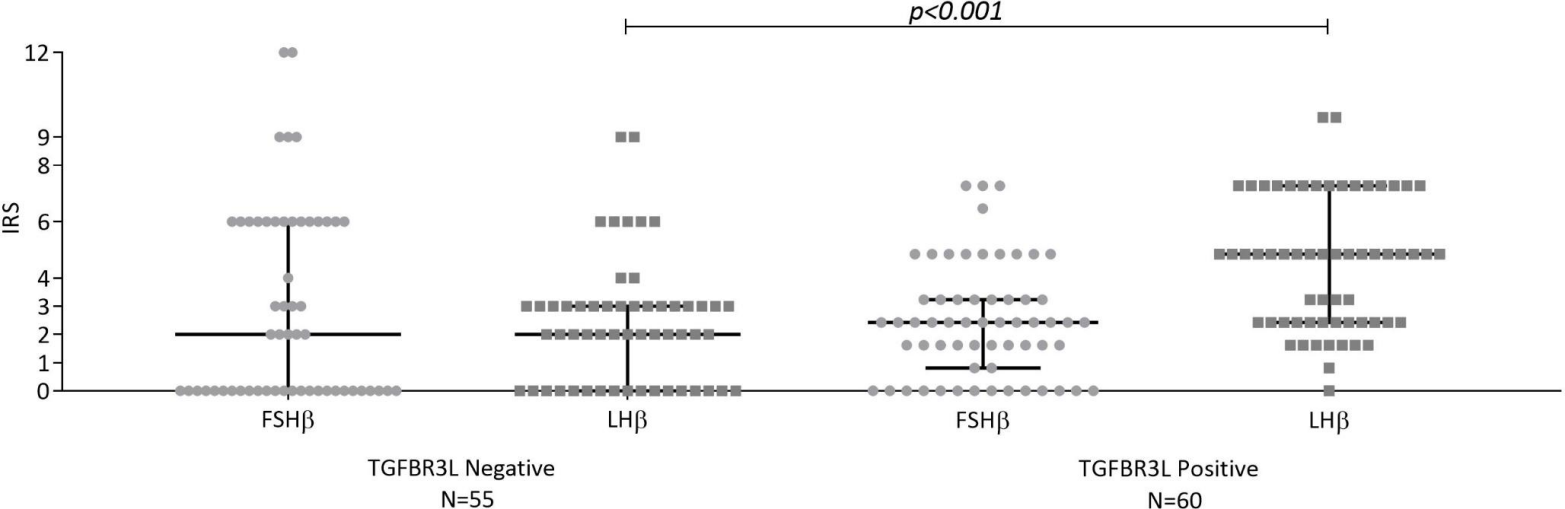
**Quantiative assessment of TGFBR3L and gonadotropin immunostaining in gonadotroph PitNETs**. TGFBR3L was associated with IRS LHβ (p < 0.001), but not IRS FSHβ (p = 0.32). Data is presented as median and interquartile range (error bars). Statistical significance was determined using Mann-Whitney U test

Presence of TGFBR3L was associated with a higher IRS of LHβ in gonadotroph NF-PitNETs (median 2 [IQR 0–3]) in TGFBR3L negative and median 6 [IQR 3–9]) in TGFBR3L positive tumours, p < 0.001). This was also the case when considering males (LHβ IRS median 2 [IQR 1–3] in the negative and median 6 [IQR3-9] in TGFBR3L positive tumours, p < 0.001) and females (LHβ IRS median 2 [IQR 0–3] in negative and median 6 [IQR3-9] in positive TGFBR3L tumours, p < 0.001). The difference in LHβ IRS between TGFBR3L negative and positive gonadotroph tumours remained significant when investigating postmenopausal women alone and in males without testosterone substitution (data not shown). Figure 2 presents the distribution of FSHβ and LHβ in TGFBR3L negative and positive tumours.

Presence of TGFBR3L staining was not associated with FSHβ staining (median 2 [IQR 0–6] and median 3 [IQR1-4] in TGFBR3L negative and positive tumours, respectively, p = 0.32) in gonadotroph NF-PitNETs. This was also true when analysing males and females separately (data not shown). When investigating the different subgroups of gonadotroph tumours we found that none of the tumours staining for FSHβ alone (n = 6) and only one of the tumours not staining for neither FSHβ nor LHβ (n = 12) presented positive for TGFBR3L. However, 46.4% of the LHβ positive tumours and 66.7% of both FSHβ and LHβ positive tumours presented positive TGFBR3L staining (p < 0.001). We found that the groups staining for either FSHβ, LHβ or both more often presented staining for TGFBR3L than tumours not staining for the gonadotropins. There was no difference in preoperative cavernous sinus invasion nor tumour volume between the four different gonadotroph sub cohorts (data not shown).

### Plasma gonadotropins

**Fig. 3:**
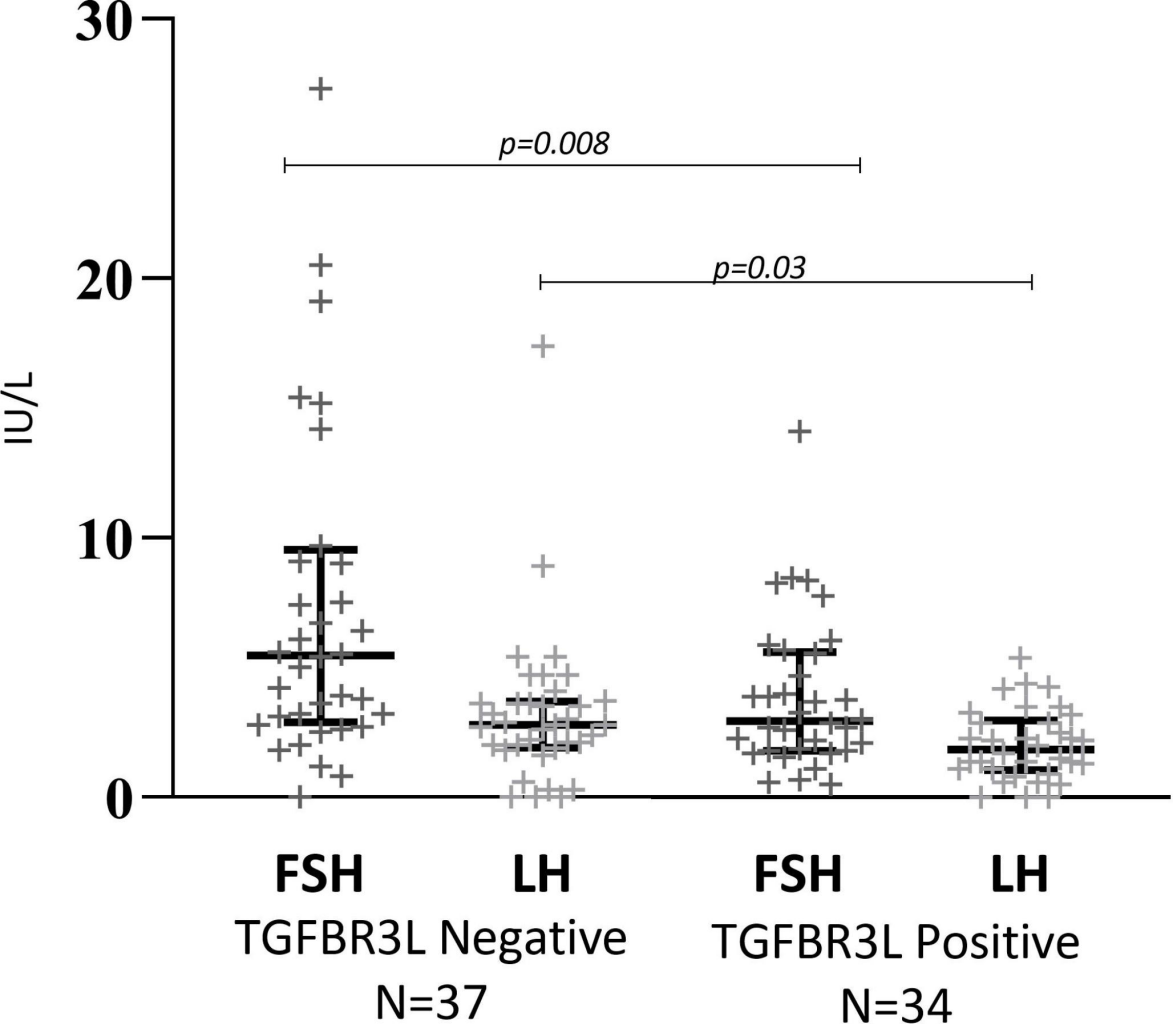
**TGFBR3L immunostaining and P-FSH and P-LH in males with gonadotroph PitNETs**. TGFBR3L was negatively associated to plasma FSH (P-FSH) (p = 0.008) and P-LH (p = 0.03) in male patients. The Y-axis represent the plasma values of FSH and LH in IU/L. Data is presented as median and interquartile range for TGFBR3L negative and positive tumours. Statistical significance was determined using Mann-Whitney U test

In males with gonadotroph NF-PitNETs, we found a negative association between the presence of TGFBR3L staining and circulating FSH (median 5.5 IU/L [IQR 2.9–9.6] in TGFBR3L negative and median 3.0 [IQR 1.8–5.6] in TGFBR3L positive tumours, p = 0.008), and P-LH (median 2.8 IU/L [IQR 1.9–3.7] in TGFBR3L negative and median 1.8 [IQR 1.1-3.0] in TGFBR3L positive tumours, p = 0.03) (Fig. 3). The association remained when we excluded males with testosterone substitution (data not shown). In males, P-FSH correlated with FSHβ staining (R = 0.239, p = 0.04), while P-LH did not correlate with LHβ staining (R=-0.191, p = 0.11).

In females with gonadotroph NF-PitNETs, TGFBR3L staining was not associated with levels of circulating P-FSH (median 5.4 IU/L [IQR 3.8–13.6] and median 6.8 [IQR 3.9–9.9], in TGFBR3L negative and positive tumours respectively, p = 0.74) or P-LH (median 1.5 IU/L [IQR 0.7–7.8] and median 1.9 [IQR 1.3–4.8] in TGFBR3L negative and positive tumours respectively, p = 0.9). We found no association between TGFBR3L and plasma gonadotropins in the group of postmenopausal women alone (data not shown). In females, tumour staining for gonadotropins was not associated with the plasma gonadotropins (data not shown).

### Tumour invasiveness and volume

There was no association between the presence of TGFBR3L and preoperative cavernous sinus invasion in gonadotroph NF-PitNETs. Fifteen (27%) of the TGFBR3L negative tumours and 17 (28%) TGFBR3L positive tumours were considered invasive, p = 0.89). The presence of TGFBR3L was associated with a higher preoperative tumour volume, median 4769 mm^3^ (IQR 3299–7896) in TGFBR3L negative tumours and median 7163 mm^3^ (IQR 4719–9511) in TGFBR3L positive tumours, p = 0.03. The tumour volume of gonadotroph NF-PitNETs was not associated with levels of circulating gonadotropins when considering both genders (P-FSH R=-0.015, p = 0.96 and P-LH R=-0.07, p = 0.48), or when considering males or females separately (data not shown). The circulating levels of P-prolactin was not associated with levels of P-FSH, P-LH or with TGFBR3L staining in any subgroup of patients (not shown).

### Transforming growth factor beta receptor 3

Considering all subtypes of NF-PitNETs, TGFBR3 staining was negative (staining score 0) in two thirds of the tumours (staining missing in 3 tumours). Of the gonadotroph tumours, 78.1% (n = 89) were negative for TGFBR3, 20.2% (n = 23) of tumours stained positive in 1–10% of cells and only two tumours had ≥ 10% positive staining cells.

Fifteen (28%) of the TGFBR3L negative gonadotroph tumours and 10 (17%) of the TGFBR3L positive tumours also stained positive for TGFBR3 (p = 0.18). There was no association between the presence of TGFBR3 and P-FSH (median 5.0 IU/L (IQR 2.7–8.5) and median 5.7 (IQR 2.3–8.9) in TGFBR3 negative and positive tumours, respectively, p = 0.80) or P-LH (median 2.1 IU/L (IQR1.1-3.6) and median 2.9 (IQR 1.0-4.6) in TGFBR3 negative and positive tumours, respectively, p = 0.70). The gonadotroph tumours staining for both TGFBR3L and TGFBR3 (n = 10) did not deviate from the rest of the gonadotroph cohort in regards to age, gender, staining for FSH and LH, tumour volume or cavernous sinus invasion (data not shown).

## Discussion

In this prospective study we found that TGFBR3L staining was presented selectively in gonadotroph PitNETs, in accordance with previous retrospective findings [26] and was positively associated with LHβ staining. Furthermore, TGFBR3L staining was not related to invasive tumour growth into the cavernous sinus, age or gender. We found a weak association between TGFBR3L and preoperative tumour volume. In addition, there was a negative association between TGFBR3L tumour staining and circulating gonadotropins in males, also when excluding patients receiving sexual hormone substitution. Finally, no relation between TGFBR3L and TGFBR3 staining was found, the latter being absent or expressed at a low level in the majority of gonadotroph tumours.

As compared to our previous study performed in a retrospective cohort of PitNETs tissue microarrays, where we found that one third of the gonadotroph PitNETs were TGFBR3L positive [26], the percentage of positive tumours in this study was slightly higher (i.e. one half). This might be due to the difference in assessing tissue microarray vs. whole tumour slides where larger areas of tumour tissue are examined, as a degree of intra-tumour heterogeneity in the TGFBR3L staining. Another explanation could be that the two cohorts of gonadotroph tumours may differ by other means due to inclusion bias, leading to a real difference in the TGFBR3L expression. In addition, we found that in human gonadotroph PitNETs, TGFBR3L staining was more related to LHβ than to FSHβ staining. The previously described association to FSHβ did not reach statistical significance in the present cohort. Here, we used a different system of grading the IHC staining of gonadotropins. However, there was no association between TGFBR3L and FSHβ when applying the grading based solely on positively staining cells used in the earlier study (data not shown). Also, no tumours exclusively staining for FSHβ, and not LHβ, presented TGFBR3L staining.

The hormone negative but SF1 positive gonadotroph tumours were more often TGFBR3L negative than tumours staining for LHβ alone or both FSHβ and LHβ. Raverot et al. have previously investigated the different subgroups of gonadotroph NF-PitNETs, and found, in concordance with our data, that the combined FSHβ and LHβ tumours were the most frequent. However, they did not include hormone-negative gonadotroph tumours in their cohort [35]. This group of gonadotroph tumours were previously, before routine staining for transcription factors, included into the group of null-cell PitNETs [36]. The over representation of TGFBR3L negative tumours among the hormone negative gonadotroph NF-PitNETs might point to these tumours being less differentiated than the hormone positive tumours.

The positive relation between TGFBR3L and the gonadotropin expression in the gonadotroph PitNETs supports the observation that TGFBR3L is a gonadotroph specific protein. In addition, due to the amino acid sequence similarity to the TGFBR3, described as an inhibin A co-receptor in mice, it is reasonable to hypothesize that TGFBR3L might be involved in the regulation of gonadotropin secretion. Indeed, this was confirmed in a recent study demonstrating that TGFBR3L functioned as an inhibin B co-receptor [23]. In TGFBR3L knockout mice, a (non-significant) increase in circulating FSH and pituitary *Fshb* mRNA, but no change in circulating LH or *Lhb* expression as compared to wild type littermates was shown [23]. Similarly, previous studies of gonadotroph cells have shown that inhibins suppress *Fshb* mRNA levels, FSHβ protein synthesis and FSH secretion, while they suppress LH secretion without affecting *Lhb* mRNA or LHβ protein synthesis [37, 38]. This may explain the positive association between TGFBR3L and LHβ staining in our present study.

Several studies have found that gonadotroph PitNETs typically produce and secrete FSH more frequently than LH, both in vivo and in vitro [9, 14, 15]. P-FSH has also been shown to correlate with FSHβ staining, while P-LH was not related to LHβ staining [16]. Taken together, these studies suggest that some of the gonadotropins produced in gonadotroph PitNETs are released, but that LH less frequently enters the circulation [9, 14–16]. This seems to occur even though FSHβ and LHβ show similar staining patterns within the gonadotroph tumours, as found in the present study. We found a negative association between TGFBR3L and circulating gonadotropins in males and in the entire population when excluding postmenopausal women and patients receiving sexual hormone replacement therapy. Although the relation of TGFBR3L with gonadotropin secretion might be gender dependent, the unsystematically recording and the cyclic variations in women makes this conclusion uncertain. Only a few premenopausal women were reported to have a regular menstrual cycle (n = 5).

We have previously described an inverse relationship between TGFBR3L protein expression and membranous E-cadherin staining in the gonadotroph PitNETs. Furthermore, tumours positive for TGFBR3L also had a nuclear accumulation of E-cadherin suggesting a possible role of TGFBR3L in tumour aggressiveness [26]. Increased expression of TGFBR3L has also been associated with development of neuroblastomas, and a more aggressive phenotype [39]. We could not prove a relation between the staining for TGFBR3L and preoperative cavernous sinus invasion, and the relation to tumour volume should be interpreted cautiously. All included tumours were macroadenomas admitted to surgery and the majority due to growth or compression symptoms, hence the preoperative tumour volume might present as a weak marker of tumour aggressiveness. Postoperative MRI follow-up studies could more precisely unveil differences in tumour aggressiveness based on the presence of TGFBR3L. The weakly positive nature of the TGFBR3L staining may also affect the association with tumour volume.

In our study, TGFBR3 staining was absent or very low in the majority of gonadotroph tumours, although several studies have shown that TGFBR3 was expressed in gonadotroph cells of murine models suggesting an important role in gonadotropin regulation [40, 41]. Another study did not find TGFBR3 (staining or gene-expression) to be increased in gonadotroph cells in rat pituitaries [42, 43]. Furthermore, the same study found no association between TGFBR3 and P-FSH or FSHβ staining, suggesting that it has other functions than being an inhibin co-receptor [42, 43]. In the present study, TGFBR3 was not associated with TGFBR3L or gonadotropin staining, results that somehow limit its possible role in gonadotroph tumour biology.

### Limitation

This study was based on IHC, biochemical and radiological data. Therefore, the function of TGFBR3L and its downstream signalling could not be determined. The plasma levels of FSH, LH and prolactin were collected from routine blood sampling taken at different time points prior to surgery, and are therefore not systematically collected. Plasma levels of inhibin B were unfortunately not available.

## Conclusion

TGFBR3L is selectively expressed in the gonadotroph cells, and is strongly associated with LHβ staining in all patients, but inversely with P-LH in males, suggesting that TGFBR3L may be involved in the release of LH from gonadotroph PitNETs. We found no association between the IHC staining for TGFBR3L and TGFBR3 in gonadotroph tumours.

## Electronic supplementary material

Below is the link to the electronic supplementary material.

Supplementary Material 1

## Data Availability

The datasets generated during and/or analysed during the current study are available from the corresponding author on reasonable request.
