# Supplementary figures and images for: TGFBR3L is associated with gonadotropin production in non-functioning gonadotroph pituitary neuroendocrine tumours
Source: Pituitary. 2023 Mar 23;26(2):227–36. doi: 10.1007/s11102-023-01310-x (PMC10247857; doi:10.1007/s11102-023-01310-x)

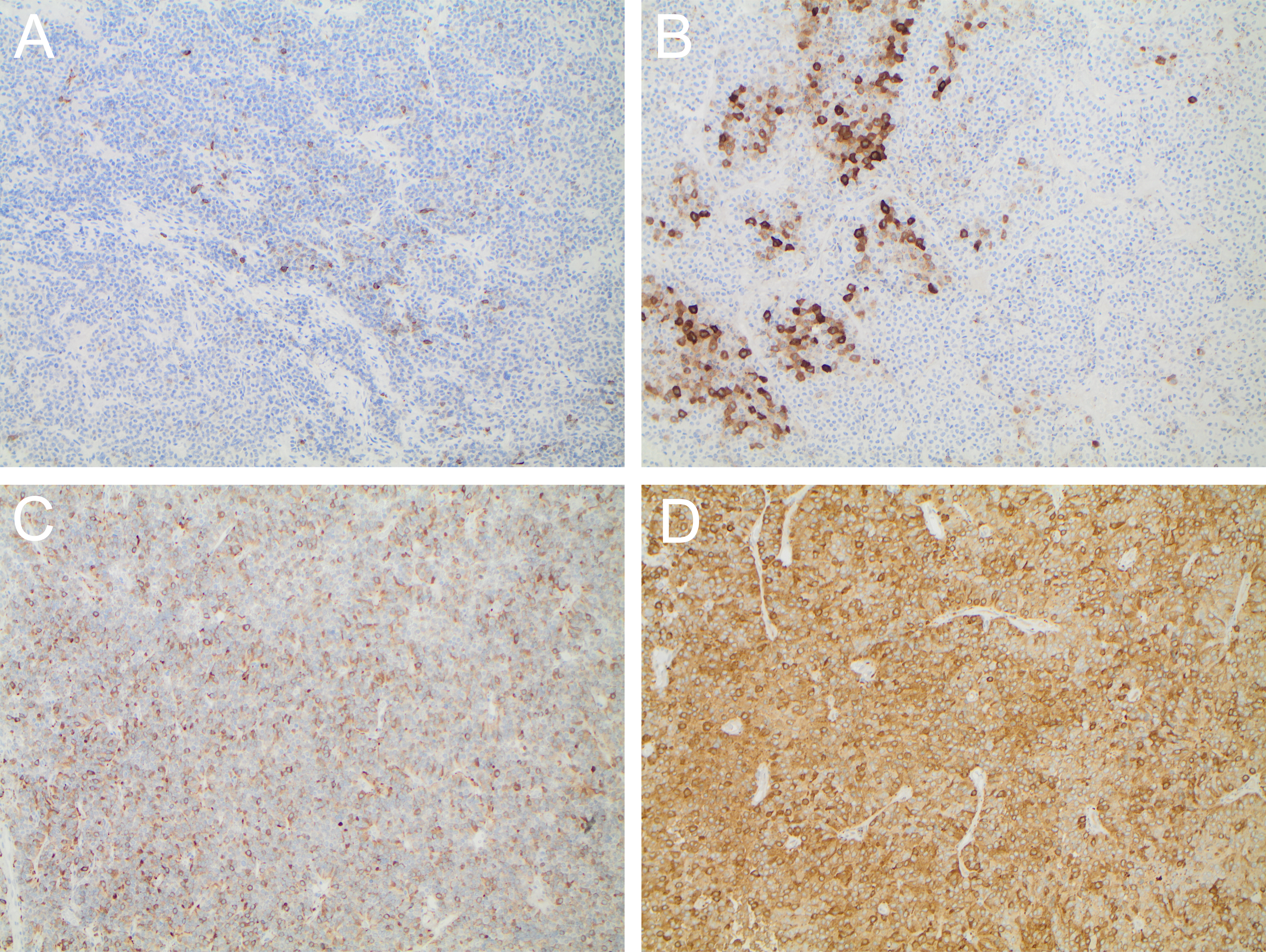

Supplement: Supplementary file 1 — Supplementary Material 1 [file 11102_2023_1310_MOESM1_ESM.tif]
